# First‐Degree Family History of Diabetes Is Associated With the Presence of Depressive Symptoms Independent of Lifestyle Risk Factors and Cardiometabolic Risk Factors

**DOI:** 10.1111/1753-0407.70139

**Published:** 2025-08-17

**Authors:** Mengying Chen, Huimin Xia, Yaohui Yu, Yuhua Wang, Wei Chen, Enyu Lou, Zhezhe Tang, Lijuan Yang, Shengjie Ge, Bo Yang, Xuejiang Gu, Xiang Hu

**Affiliations:** ^1^ Department of Endocrine and Metabolic Diseases The First Affiliated Hospital of Wenzhou Medical University Wenzhou China; ^2^ School of the First Clinical Medical Sciences (School of Information and Engineering) Wenzhou Medical University Wenzhou China; ^3^ Department of Preventive Medicine, School of Public Health and Management Wenzhou Medical University Wenzhou China; ^4^ Institute of Lipids Medicine Wenzhou Medical University Wenzhou China

**Keywords:** depressive symptoms, diabetes, first‐degree family history of diabetes

## Abstract

**Background:**

Co‐occurrence of depression and diabetes is a prototypical example of mental‐physical comorbidity. This study aims to investigate the association between first‐degree family history of diabetes (FHD) and the presence of depressive symptoms.

**Methods:**

The present work was one part of the baseline survey from the REACTION study. First‐degree FHD was defined as having one or more first‐degree relatives with diabetes. The Patient Health Questionnaire‐9 was administered to detect the presence of depressive symptoms with its score ≥ 5. Logistic regression analyses were performed to determine the association between first‐degree FHD and the presence of depressive symptoms.

**Results:**

A total of 4804 participants were enrolled in the present study. Individuals with first‐degree FHD were more likely to suffer from depressive symptoms compared with those without first‐degree FHD (7.2% versus 4.9%, *p* = 0.004). The odds ratio (OR) of depressive symptoms was increased by 49.8% with the presence of first‐degree FHD after adjustment of gender, age, socioeconomic factors, lifestyle risk factors, and cardiometabolic risk factors (*p* = 0.007). There were no significant interactions of gender, age, each socioeconomic factor, lifestyle risk factor, and cardiometabolic risk factors on the association between first‐degree FHD and the presence of depressive symptoms, respectively (all *p* > 0.05).

**Conclusions:**

First‐degree FHD was associated with depressive symptoms independent of socioeconomic factors, lifestyle risk factors, and cardiometabolic risk factors. Genetic background might mainly contribute to the familial aggregation of depressive symptoms in individuals with first‐degree FHD, who should be paid early attention to their mental health.

## Graphical abstract

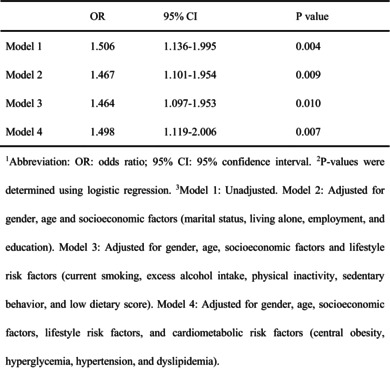

**Summary**

- First‐degree family history of diabetes (FHD), especially parental FHD, was independently linked to increased depressive symptoms, with no discernible interaction observed with socioeconomic factors or lifestyle risk factors or cardiometabolic risk factors.
- This underscores the potentially prominent role of genetic factors in the familial clustering of depressive symptoms among individuals with first‐degree FHD.
- Early monitoring of the mental well‐being of individuals with FHD, irrespective of their lifestyle risk factors, holds promise in mitigating the risk of depression in diabetic patients.

## 1 Introduction

The concurrent occurrence of diabetes and depression exemplifies a prototypical instance of psychosomatic comorbidity [1]. Epidemiological data indicate that a minimum of one‐third of individuals with diabetes will develop depression, whereas individuals with depression face a 37% elevated risk of developing diabetes [2]. This comorbidity contributes significantly to heightened morbidity, mortality, and diminished health‐related quality of life, culminating in an increased disease burden and elevated healthcare costs [3, 4].

Growing evidence suggests that genetic factors play a pivotal role in the onset and progression of diabetes and depression. Multiple twin studies demonstrate a clear genetic overlap [5, 6]. Genes involved in the neuroendocrine cortisol pathway, such as corticotropin‐releasing hormone receptor genes (CRHR1 and CRHR2), adrenocorticotropic hormone receptor or melanocortin receptor genes (MC1R‐MC5R), glucocorticoid receptor genes (GCR), and mineralocorticoid receptor gene (MCR), have been implicated in the comorbidity of diabetes and depression [7, 8, 9]. In addition, both disorders share biological mechanisms linking, including heightened activation of the hypothalamic–pituitary–adrenal (HPA) axis, chronic inflammation, excessive activation of innate immunity response, dysrhythmias, and insulin resistance [10, 11, 12].

Individuals with a first‐degree family history of diabetes (FHD) were defined as having at least one parent, sibling, or child with diabetes [13]. They not only share a genetic background with diabetic patients, but also live in a similar family environment [13]. Therefore, both the genetic background and family environment might contribute to the increased risk of depression in individuals with first‐degree FHD. Genetic research has identified shared genetic variations between diabetes and depression, implying a genetic correlation [11, 14]. Two clinical studies, one in Korea [15] and another in the United States [16], have also found a positive link between the diabetes status of family members and the presence of depressive symptoms.

Yet, limited clinical studies impede clear conclusions, and there remains insufficient clinical evidence in the Chinese population. To address the role of FHD in the development of depression, we aimed to investigate if the first‐degree FHD was independently associated with the presence of depressive symptoms. Additionally, we examined the impact of socioeconomic factors, lifestyle risk factors, and cardiometabolic risk factors on this association.

## 2 Methods

### 2.1 Study Population

The current study was based on the baseline survey from the REACTION study in China, which was conducted between 2011 and 2012 in 25 local communities to investigate the association between diabetes and cancer [17]. The present study participants were recruited from four communities in Wenzhou City. Participants with the following characteristics were excluded: severe liver or kidney dysfunction, severe cardiovascular or cerebrovascular disease, tumor, acute infection, hyperthyroidism or hypothyroidism, and psychiatric disorder. In addition, participants who were unable to provide information on family history of diabetes or missed the questionnaire were also excluded. Finally, a total of 4804 participants were included in the current analysis (Figure S1).

This study was conducted in accordance with the principles of the Declaration of Helsinki and approved by the Ruining Hospital Ethics Committee, Shanghai Jiao Tong University School of Medicine (201114RHEC). All subjects signed the informed consent before participating in the study.

### 2.2 Data Collection

All participants completed standardized questionnaires which were administered by professional physicians during personal interviews. The questionnaire mainly included socio‐demographic characteristics (marital status, living alone, employment, and education level), lifestyle risk factors (smoking, alcohol, physical activity, sedentary behavior, and dietary factors), as well as medical history and family history of diabetes. The definitions of the above factors have been described in previous studies [17, 18].

Physical examinations including weight, height, waist circumference (WC) and blood pressure were conducted by trained staff. Weight and height were measured by participants with light clothing and no shoes. Body mass index (BMI) was calculated as weight (kg)/height^2^ (m^2^). WC was measured in standing participants along the costal margin and the upper margin of the iliac crest using a tape measure. Blood pressure was measured with a standard sphygmomanometer every 5 min of rest in a seated position, and the mean of three consecutive measurements was taken into final analysis. After fasting at least 8 h, blood samples were collected, including fasting plasma glucose (FPG), glycated hemoglobin A1c (HbA1c), total cholesterol (TC), triglyceride (TG), high‐density lipoprotein cholesterol (HDL‐C), and low‐density lipoprotein cholesterol (LDL‐C). Participants without a prior history of diabetes received a standard 75‐g oral glucose tolerance test (OGTT), while those with a history of diabetes were administered a standard meal containing 100 g of carbohydrates [17]. The 2‐h plasma glucose (2hPG) was measured 2 h after the administration of the 75‐g OGTT or the steamed bread meal test. All of the above biochemical indicators were detected under strict quality control.

### 2.3 Definitions

First‐degree FHD was defined as having at least one diabetic patient in parents, siblings, or children [13]. Patient Health Questionnaire‐9 (PHQ‐9) was applied to assess depressive symptoms, and PHQ‐9 score ≥ 5 was defined as the presence of depressive symptoms [19]. Marital status was categorized as married/cohabitant or other (including never married, widowed, separated or divorced). Current living status was classified as living alone or not. Employment status was categorized as employed or unemployed/retired. Education level was stratified into two categories: Junior high school education or below, and high school education or above. Participants who smoked once a day or seven times per week regularly during the past 6 months were defined as current smokers. Participants who drank regularly once a week in the past 6 months were considered as current drinkers. Physical activity was estimated using the short form of the International Physical Activity Questionnaire (IPAQ) and participants taking moderate‐intensity exercise for ≥ 150 min per week or vigorous‐intensity exercise for ≥ 75 min per week were considered as physically active [20]. Sedentary time ≥ 30 h per week was defined as the presence of sedentary behavior. Dietary scores were derived from seafood, fruits and vegetables, soy protein, and sugar‐sweetened beverages, with a score of less than 2 defined as a low dietary score, indicating a less healthy diet [17]. BMI ≥ 25 kg/m^2^ was defined as overall overweight/obesity [21]. WC ≥ 85 cm in women and ≥ 90 cm in men was considered as central overweight/obesity [22]. Hypertension was defined as systolic blood pressure (SBP) ≥ 140 mmHg and/or diastolic blood pressure (DBP) ≥ 90 mmHg and/or on antihypertensive treatment [23]. Hyperglycemia was defined as FPG ≥ 6.1 mmol/L and/or 2hPG ≥ 7.8 mmol/L and/or HbA1c ≥ 6.5%, and/or previous diagnosis for diabetes [24]. Dyslipidemia was defined as TC ≥ 5.18 mmol/L, TG ≥ 1.7 mmol/L, LDL‐C ≥ 3.37 mmol/L, HDL‐C < 1.04 mmol/L, or on lipid‐lowering therapy [25].

### 2.4 Statistical Analysis

Continuous variables were expressed as mean ± standard deviation (SD) for normal distribution or median with interquartile range (IQR) for skewed distribution. Categorical variables were presented as numbers with percentages. Comparisons between groups were measured by the student's *t* test for continuous variables with normal distribution or the Mann–Whitney U test for continuous variables with skewed distribution and chi‐square test for categorical variables. Logistic regression analysis was conducted to assess the association between first‐degree FHD or parental FHD and the presence of depressive symptoms as well as the interaction of socioeconomic factors (marital status, living alone, employment, and education), lifestyle risk factors (current smoking, excess alcohol intake, physical inactivity, sedentary behavior, and low dietary score) and cardiometabolic risk factors (central obesity, hyperglycemia, hypertension, and dyslipidemia) with this association. Sensitivity analyses were conducted to further assess the robustness of the association by performing logistic regression in a subgroup of participants with data on sleep duration. Statistical analysis was performed using IBM SPSS version 26.0 (IBM Corp., Armonk, NY, United States). All *p* values were two‐tailed, and *p* < 0.05 was considered statistically significant.

## 3 Results

### 3.1 Clinical Characteristics of the Study Participants

A total of 4804 participants with an average age of 58.74 ± 8.43 years (age range: 39.50–80.23 years) were included, with 1006 participants with first‐degree FHD and 3798 participants without first‐degree FHD (Table 1). Compared with individuals without first‐degree FHD, individuals with first‐degree FHD were younger (*p* < 0.001) and had higher levels of FPG, 2hPG, HbA1c, and TG and lower levels of WC and SBP. In addition, the proportions of women, individuals with diabetes, dyslipidemia, and sedentary behaviors in subjects with first‐degree FHD were higher than those without first‐degree FHD (all *p* < 0.01).

**TABLE 1:** Characteristics of the study population.

Variable	Total	Individuals with FHD	Individuals without FHD	*p*
Participants (*n*)	4804	1006	3798	
Socio‐demographic factors				
Gender (*n*, %)				< 0.001
Men	1527 (31.8)	275 (27.3)	1252 (33.0)	
Women	3277 (68.2)	731 (72.7)	2546 (67.0)	
Age (*n*, %)				< 0.001
< 60	2606 (54.2)	632 (62.8)	1974 (52.0)	
≥ 60	2198 (45.8)	374 (37.2)	1824 (48.0)	
Marital status (*n*, %)				0.744
Married/cohabitant	4308 (89.7)	899 (89.5)	3409 (89.8)	
Others	493 (10.3)	106 (10.5)	387 (10.2)	
Living alone (*n*, %)				0.532
Yes	297 (6.2)	58 (5.8)	239 (6.3)	
No	4498 (93.8)	947 (94.2)	3551 (93.7)	
Employment (*n*, %)				0.755
Employed	956 (20.2)	196 (19.9)	760 (20.3)	
Unemployed/retired	3769 (79.8)	790 (80.1)	2979 (79.7)	
Education level (*n*, %)				0.017
Junior high school education or below	3997 (83.4)	812 (80.9)	3185 (84.0)	
High school education or above	797 (16.6)	192 (19.1)	605 (16.0)	
Lifestyle risk factors				
Current smoking (*n*, %)	668 (13.9)	134 (13.3)	534 (14.1)	0.546
Excess alcohol intake (*n*, %)	604 (12.6)	125 (12.4)	479 (12.6)	0.874
Physical inactivity (*n*, %)	3715 (77.3)	762 (75.7)	2953 (77.8)	0.177
Sedentary behavior (*n*, %)	1997 (41.6)	480 (47.7)	1517 (39.9)	< 0.001
Low dietary score (*n*, %)	1519 (31.6)	304 (30.2)	1215 (32.0)	0.283
Cardiometabolic risk factors				
Overall overweight/obesity (*n*, %)	1849 (38.5)	395 (39.3)	1454 (38.3)	0.570
Central overweight/obesity (*n*, %)	2293 (47.7)	458 (45.5)	1835 (48.3)	0.115
Hyperglycemia (*n*, %)	2363 (49.2)	584 (58.1)	1779 (46.8)	< 0.001
Hypertension (*n*, %)	2440 (50.8)	496 (49.3)	1944 (51.2)	0.289
Dyslipidemia (*n*, %)	3803 (79.2)	833 (82.8)	2970 (78.2)	0.001
Laboratory indicators				
BMI (kg/m^2^)	24.1 (22.1–26.3)	24.2 (22.1–26.2)	24.1 (22.1–26.3)	0.832
WC (cm)	86.0 (80.0–92.0)	85.2 (79.9–91.0)	86.0 (80.0–92.0)	0.017
SBP (mmHg)	133.0 (120.0–146.3)	132.3 (119.7–144.7)	133.3 (120.0–147.0)	0.034
DBP (mmHg)	79.7 (72.3–86.7)	79.3 (72.0–86.3)	79.7 (72.3–87.0)	0.225
FPG (mmol/L)	5.5 (5.1–6.2)	5.7 (5.2–6.8)	5.4 (5.0–6.0)	< 0.001
2hPG (mmol/L)	7.3 (6.0–10.0)	7.9 (6.3–11.5)	7.2 (5.9–9.7)	< 0.001
HbA1c (%)	5.8 (5.5–6.2)	5.9 (5.6–6.6)	5.8 (5.5–6.2)	< 0.001
TC (mmol/L)	5.4 (4.7–6.1)	5.4 (4.7–6.1)	5.4 (4.7–6.1)	0.188
TG (mmol/L)	1.5 (1.1–2.2)	1.6 (1.1–2.2)	1.5 (1.1–2.1)	0.011
HDL‐c (mmol/L)	1.4 (1.2–1.6)	1.4 (1.2–1.6)	1.4 (1.2–1.6)	0.161
LDL‐c (mmol/L)	3.2 (2.6–3.7)	3.2 (2.6–3.7)	3.1 (2.6–3.7)	0.272

*Note:* Data were presented as median (interquartile range) or *N* (%). *p* values were determined using chi‐square test for categorical variables and Mann–Whitney U test for continuous variables.

Abbreviations: 2hPG, 2‐h plasma glucose; BMI, body mass index; DBP, diastolic blood pressure; FHD, family history of diabetes; FPG, fasting plasma glucose; HbA1c, glycated hemoglobin A1c; HDL‐c, high density lipoprotein cholesterol; LDL‐c, low density lipoprotein cholesterol; SBP, systolic blood pressure; TC, total cholesterol; TG, triglyceride; WC, waist circumference.

### 3.2 Comparison of Depressive Symptoms Between Subjects With or Without FHD

In comparison to individuals without first‐degree FHD, those with first‐degree FHD exhibited a higher prevalence of depressive symptoms (4.9% vs. 7.2%, *p* = 0.004). Participants with parental FHD showed a greater prevalence of depressive symptoms than those without parental FHD (5.0% vs. 7.1%, *p* = 0.021) (Figure S2). Similarly, depression scores were significantly higher in individuals with first‐degree FHD compared to those without first‐degree FHD (0.87 ± 1.90 vs. 1.19 ± 2.32, *p* < 0.001). Individuals with parental FHD had higher depression scores than those without parental FHD (0.90 ± 1.93 vs. 1.16 ± 2.33, *p* = 0.005) (Figure S3).

### 3.3 The Association of Depressive Symptoms With First‐Degree FHD

Association between first‐degree FHD and the presence of depressive symptoms was shown in Table 2. First‐degree FHD was associated with increased odds of depressive symptoms (OR = 1.506, 95% CI: 1.136–1.995, *p* = 0.004). This association remained statistically significant with multiple adjustments of age, gender, socioeconomic factors, lifestyle risk factors, and cardiometabolic risk factors (OR = 1.498, 95% CI: 1.119–2.006, *p* = 0.007). Participants with parental FHD had increased odds of depressive symptoms compared with those without parental FHD (Table S1), both in crude (OR = 1.242, 95% CI: 1.062–1.451, *p* = 0.007) and multivariate‐adjusted models (OR = 1.248, 95% CI: 1.061–1.469, *p* = 0.008).

**TABLE 2:** Association between the first‐degree family history of diabetes and depressive symptoms.

	OR	95% CI	*p*
Model 1	1.506	1.136–1.995	0.004
Model 2	1.467	1.101–1.954	0.009
Model 3	1.464	1.097–1.953	0.010
Model 4	1.498	1.119–2.006	0.007

*Note:* *p* values were determined using logistic regression. Model 1: Unadjusted. Model 2: Adjusted for gender, age, and socioeconomic factors (marital status, living alone, employment, and education). Model 3: Adjusted for gender, age, socioeconomic factors, and lifestyle risk factors (current smoking, excess alcohol intake, physical inactivity, sedentary behavior, and low dietary score). Model 4: Adjusted for gender, age, socioeconomic factors, lifestyle risk factors, and cardiometabolic risk factors (central obesity, hyperglycemia, hypertension, and dyslipidemia).

Abbreviations: 95% CI, 95% confidence interval; OR, odds ratio.

### 3.4 Subgroup Analyses

**FIGURE 1:**
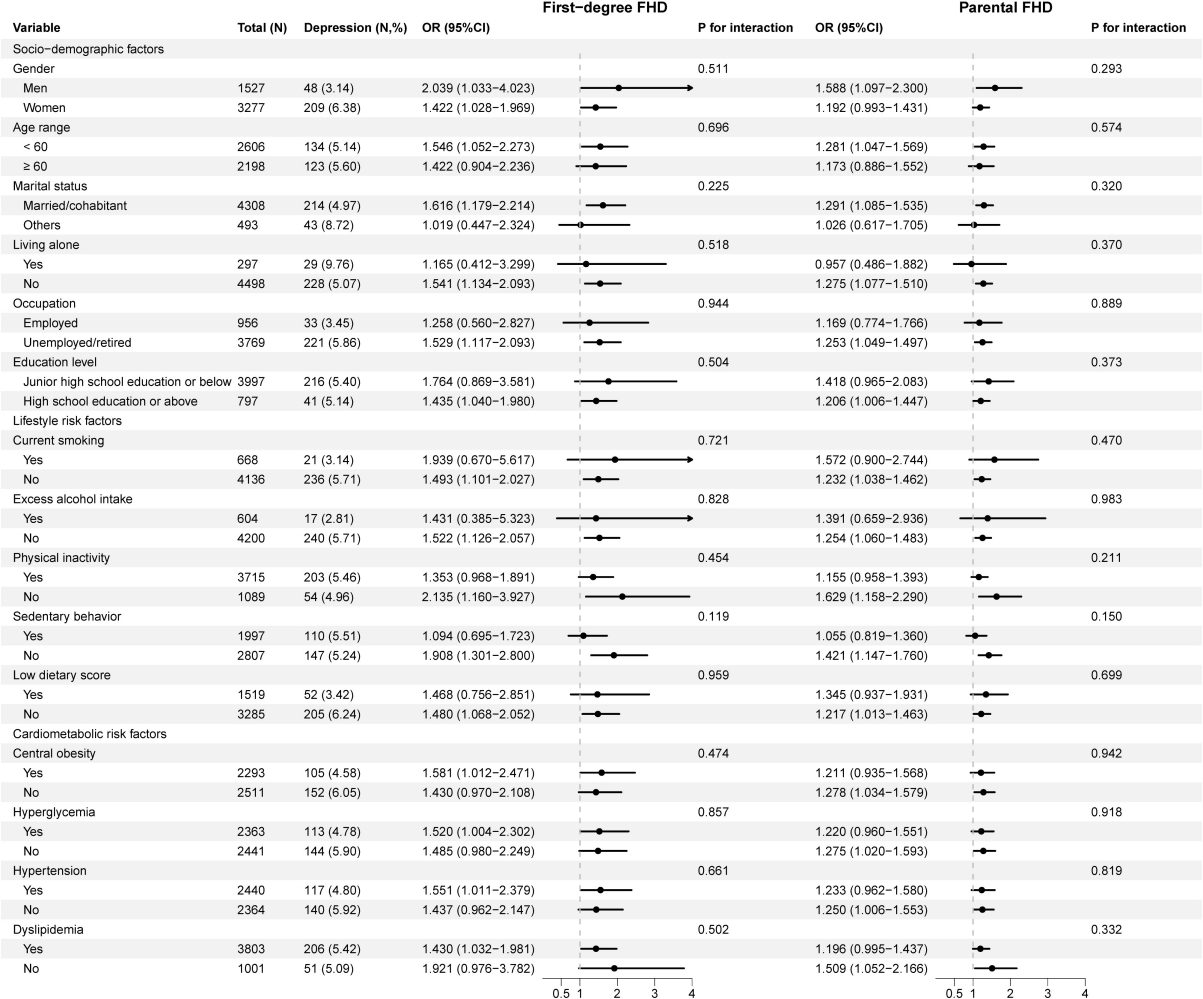
Interaction of confounding factors on the association between first‐degree family history of diabetes and the presence of depressive symptoms. Abbreviation: 95% CI, 95% confidence interval; FHD, family history of diabetes; OR, odds ratio. *p* value and *p* for interaction were determined using logistic regression. Model was adjusted for gender, age, socioeconomic factors (marital status, living alone, employment, and education), lifestyle risk factors (current smoking, excess alcohol intake, physical inactivity, sedentary behavior, and low dietary score), and cardiometabolic risk factors (central obesity, hyperglycemia, hypertension, and dyslipidemia).

Results of subgroup analyses on the association between first‐degree FHD and the presence of depressive symptoms were shown in Figure 1. First‐degree FHD had no significant interactions with gender, age, socioeconomic factors, lifestyle risk factors, and cardiometabolic risk factors on the presence of depressive symptoms. Similarly, no significant interactions were observed between parental FHD and the presence of depressive symptoms on the above aspects.

### 3.5 Sensitivity Analyses

The robustness of the association between first‐degree FHD and the presence of depressive symptoms was assessed through sensitivity analyses (Table 3). First, we repeated the main analyses using a sample of 4698 participants after excluding those without data on sleep duration (*N* = 106). After adjusting for age, gender, socioeconomic factors, lifestyle risk factors, cardiometabolic risk factors, and sleep duration (≤ 6 h, 6–8 h, > 8 h), both first‐degree FHD (OR = 1.458, 95% CI: 1.087–1.955, *p* = 0.012) and parental FHD (OR = 1.228, 95% CI: 1.043–1.447, *p* = 0.014) were significantly associated with the presence of depressive symptoms. Second, we further excluded individuals currently taking hypnotics within the above study population with available sleep duration data, and the associations of first‐degree and parental FHD with depressive symptoms were almost not altered.

**TABLE 3:** Sensitivity analyses on association between the first‐degree family history of diabetes and the presence of depressive symptoms.

	Participants (*n*)	Depressive symptoms (*n*, %)	OR	95% CI	*p*
Excluding missing data of sleep duration					
First‐degree FHD					
Crude model	993	72 (7.25%)	1.505	1.135–1.995	0.005
Muli‐adjusted model			1.458	1.087–1.955	0.012
Parental FHD					
Crude model	704	51 (7.24%)	1.242	1.062–1.451	0.007
			1.228	1.043–1.447	0.014
Multi‐adjusted model					
Further excluding individuals with hypnotics use					
First‐degree FHD					
Crude model	972	64 (6.58%)	1.494	1.110–2.012	0.008
			1.472	1.080–2.005	0.014
Multi‐adjusted model					
Parental FHD					
Crude model	691	44 (6.37%)	1.224	1.038–1.444	0.017
Multi‐adjusted model			1.219	1.025–1.449	0.025

*Note:* *p* values were determined using logistic regression. Multi‐adjusted model was adjusted for gender, age, socioeconomic factors (marital status, living alone, employment, and education), lifestyle risk factors (current smoking, excess alcohol intake, physical inactivity, sedentary behavior, and low dietary score), cardiometabolic risk factors (central obesity, hyperglycemia, hypertension, and dyslipidemia) and sleep duration.

Abbreviations: 95% CI, 95% confidence interval; FHD, family history of diabetes; OR, odds ratio.

## 4 Discussion

To the best of our knowledge, this is the first study to explore the relationship between first‐degree FHD and the presence of depressive symptoms in a large population of Chinese adults. Our current study showed that individuals with FHD were more prone to suffer from depressive symptoms than non‐FHD individuals. First‐degree FHD, especially the parental FHD, was independently associated with the presence of depressive symptoms, and this positive association was not interacted with socioeconomic factors, lifestyle risk factors, and cardiometabolic risk factors. Such findings suggested that genetic factors rather than lifestyle risk factors might contribute more to the familial clustering of depressive symptoms in the first‐degree relatives of diabetic patients.

The strong association between diabetes and depression has been established in previous clinical studies. In a longitudinal study of 4747 subjects, patients with diagnosed Type 2 diabetes exhibited a 1.7 times increased risk of depressive symptoms compared with subjects with normal glucose concentrations [26]. Another cohort study, which utilized the Beck Depression Inventory II (BDI‐II) to assess 458 patients with Type 1 diabetes and 546 patients without diabetes, showed that the prevalence of depression (assessed by BDI‐II cut score or antidepressant use) was significantly higher in participants with Type 1 diabetes than in those without diabetes [27]. Moreover, a cohort study involving nearly half a million Chinese adults indicated that both meeting the criteria for a major depressive episode and experiencing only depressive symptoms were associated with an increased risk of developing Type 2 diabetes [28].

Individuals with FHD and diabetic patients share a common genetic background and similar living environments, which may increase the risk of both diabetes and depression due to the shared susceptibility genes and traditional family lifestyles, such as an unhealthy diet and insufficient physical activity [29]. Additionally, the daily care of diabetic patients was primarily undertaken by family members. Caregivers of diabetic patients often experience increased stress and burden, leading to psychological anxiety and distress [30, 31]. However, the previous clinical findings on the association between FHD and depression have not yet reached consistency. JIA et al. used the NHANES database to explore the association between depression and family member diabetes status in US adults [16]. The results demonstrated that the diabetes status of family members was associated with depression, and trend analyses suggested that participants with more diabetic family members had a higher risk of depression. However, an Indian study of 481 first‐degree relatives of T2DM patients showed that the prevalence of depression in individuals with FHD was not higher than that in the general population [32]. Consistent with JIA's finding, our study showed that in the Chinese population, first‐degree FHD, especially the parental FHD, was closely related to the increased odds of depressive symptoms. Therefore, it is of great significance to pay attention to the mental health of individuals with first‐degree FHD and take effective measures to prevent depression timely.

Previous studies have indicated a close link between the development of depression and lifestyle risk factors [33]. In a meta‐analysis conducted by Zui Narita et al. the potential of physical activity in treating diabetes‐related depression was examined, revealing a significantly beneficial effect on depression [34]. Huang et al. performed a meta‐analysis of prospective studies to investigate the impact of sedentary behaviors on the risk of depression [35]. The results showed that mentally passive sedentary behaviors increased the risk of depression, whereas mentally active sedentary behaviors were not significantly associated with depression. Considering the influence of various lifestyle risk factors, including age, gender, smoking, drinking, physical activity, sedentary behavior, diet, and cardiometabolic risk factors, on the association between first‐degree FHD and the presence of depressive symptoms, the present study addressed these factors. However, our study found that the association between first‐degree FHD and the presence of depressive symptoms was independent of these lifestyle risk factors, suggesting a potential role for genetic factors in this relationship.

Subgroup analyses found that the positive association between first‐degree FHD and the presence of depressive symptoms was more pronounced in those without lifestyle risk factors and those with cardiometabolic risk factors. The presence of lifestyle risk factors may introduce confounding factors, thereby potentially distorting the relationship between first‐degree FHD and the presence of depressive symptoms. In contrast, individuals with FHD and diabetic patients share the same genetic background, with common genetic factors identified between diabetes and metabolic disorders such as obesity, hypertension, and dyslipidemia [36]. This genetic overlap might serve to strengthen the association between first‐degree FHD and the presence of depressive symptoms. According to these findings above, it is warranted to speculate that genetic background might contribute more to the association between first‐degree FHD and the presence of depressive symptoms.

The mechanism underlying the association between FHD and the presence of depressive symptoms remained unclear. Previous studies have explained to some extent the common pathogenesis between diabetes and depression, including innate inflammatory response, HPA axis dysregulation, circadian rhythm disruption, and insulin resistance [11]. Moreover, genetic factors also contribute to the comorbidity of diabetes and depression. XUAN et al. conducted a Mendelian randomization analysis in a Chinese population with high homogeneity to investigate the causal relationship between T2DM and depression, and the results supported a significant association between genetically determined T2DM and depression, rather than environmental factors [37]. In addition, Carol Kan's team separately studied the genetic overlap between T2DM and depression in twin samples from Sweden, Denmark [5], and Sri Lanka [6], all of which supported the key role of genetic effects in the association between T2DM and depression. Mutaz Amin's team has successively revealed the role of nuclear receptor subfamily 3 Group C member (NR3C1) Gene, corticotropin‐releasing hormone receptor 2 (CRHR2) Gene, and Melanocortin Receptor Genes in the comorbidity of familial diabetes and depression [8, 9, 38]. These genes are related to the HPA axis, and genetic variation may lead to the imbalance of negative feedback inhibition of the HPA axis, resulting in hypercortisolism, driving central and peripheral catecholamine secretion, and further mediating hyperglycemia and depressive emotion [39]. Additionally, transcription factor 7‐like 2 (TCF7L2) Gene, a major regulator of insulin production and processing, has been considered a prominent marker in individuals with Type 2 diabetes who have a positive family history of diabetes [40]. Recent research has found that TCF7L2 Gene also played a role in familial major depressive disorder (MDD), T2DM, and MDD‐T2DM [41]. These findings may partially explain the genetic association between FHD and depression. Further studies are needed to explore the underlying mechanisms between this association.

However, there are several limitations in our study. First, the presence of depressive symptoms was assessed by the PHQ‐9 questionnaire rather than the clinical diagnosis; therefore, patients with previous episodes of depressive symptoms were likely to be missed. Second, we did not collect the data on the use of medication, which may have affected depressive symptoms. However, we have considered the influence of prescribed use of hypnotics in the section of sensitivity analyses. Third, although we have controlled most of the covariates as much as possible, some residual confounding factors may not be taken into account. Fourth, the information in the questionnaire, such as lifestyle factors and medical history, was obtained through a self‐report, which may be affected by recall bias. Finally, this is a cross‐sectional study, and causal inferences cannot be made. Prospective cohort studies are warranted to explore the causal association between first‐degree FHD and the presence of depressive symptoms in the future.

## 5 Conclusions

In conclusion, our study demonstrated a positive association between first‐degree FHD and depressive symptoms independent of socioeconomic factors, lifestyle factors, and cardiometabolic risk factors. Genetic background might play a more important role in the familial aggregation of depressive symptoms in individuals with first‐degree FHD. Early attention was paid to the mental status of individuals with FHD, even those without lifestyle risk factors, which is beneficial to prevent against depression in diabetic patients.

## Author Contributions

Xiang Hu and Xuejiang Gu conceived and designed the study. Bo Yang contributed to the design. Xiang Hu and Xuejiang Gu conducted the study. Mengying Chen and Huimin Xia did the statistical analysis. Yaohui Yu, Yuhua Wang, Wei Chen, Enyu Lou, Lijuan Yang, Shengjie Ge, and Zhezhe Tang interpreted the data. Mengying Chen, Xiang Hu, and Bo Yang drafted the original manuscript. All authors reviewed and revised the manuscript.

## Ethics Statement

This study was conducted in accordance with the principles of the Declaration of Helsinki and approved by the Ruining Hospital Ethics Committee, Shanghai Jiao Tong University School of Medicine (201114RHEC).

## Consent

All participants signed the informed consent before participating in the study.

## Conflicts of Interest

The authors declare no conflicts of interest.

## Supporting information


**Figure S1:** Flowchart of participants selection.
**Figure S2:** Comparison of the prevalence of depressive symptoms between individuals with or without FHD.
**Figure S3:** Comparison of depression scores between individuals with or without FHD.
**Table S1:** Association between the parental family history of diabetes and depressive symptoms.

## Data Availability

The data are held in a secure, confidential database, which can only be assessed by members of the REACTION group. The data that support the findings of this study are available from the corresponding author upon reasonable request.
