# Supplementary material for: First‐Degree Family History of Diabetes Is Associated With the Presence of Depressive Symptoms Independent of Lifestyle Risk Factors and Cardiometabolic Risk Factors
Source: J Diabetes. 2025 Aug 17;17(8):e70139. doi: 10.1111/1753-0407.70139 (PMC12358737; doi:10.1111/1753-0407.70139)
Supplement: Supplementary file 1 — Figure S1: Flowchart of participants selection. Figure S2: Comparison of the prevalence of depressive symptoms between individuals with or without FHD. Figure S3: Comparison of depression scores between individuals with or without FHD. Table S1: Association between the parental family history of diabetes and depressive symptoms. [file JDB-17-e70139-s001.docx]

Supplementary Materials

Figure S1. Flowchart of participants selection.


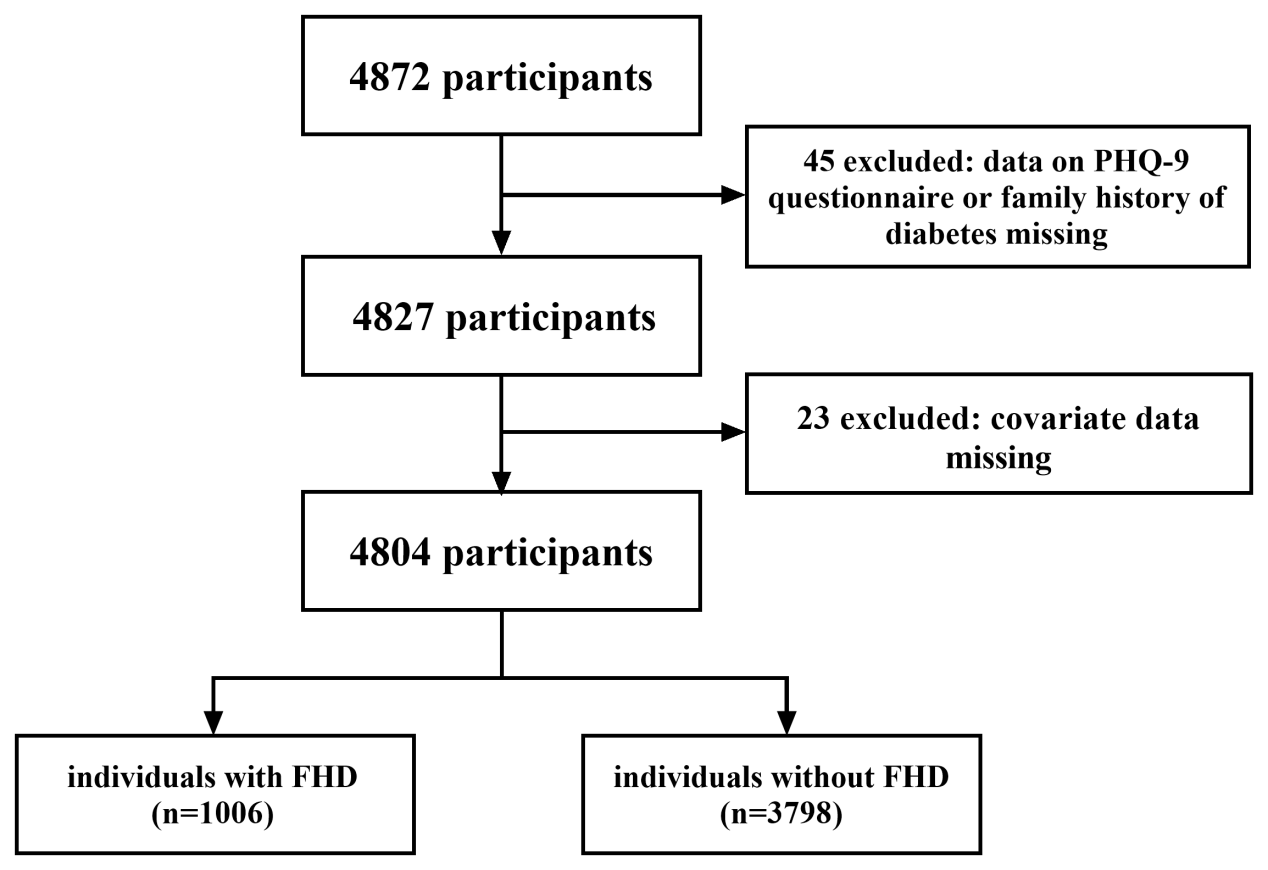


Figure S2. Comparison of the prevalence of depressive symptoms between individuals with or without FHD.


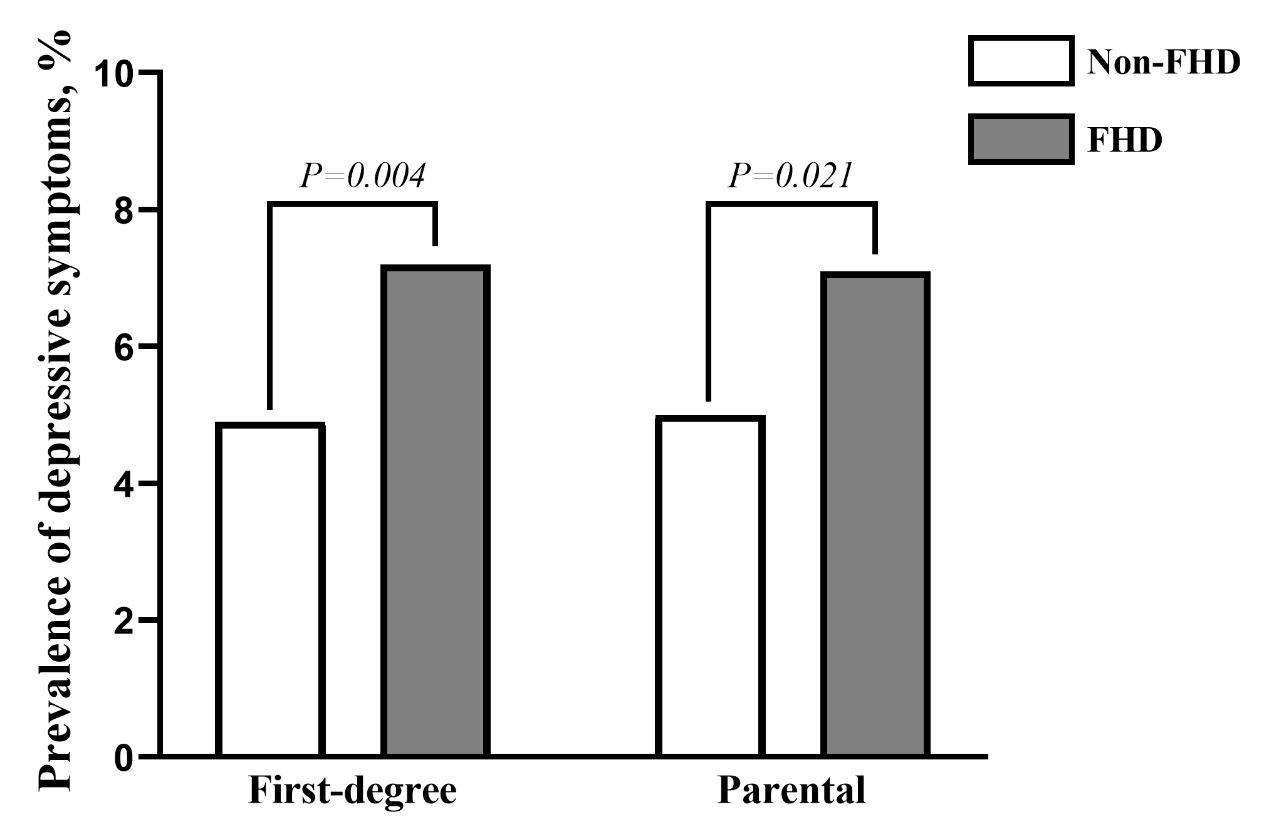


Figure S3. Comparison of depression scores between individuals with or without FHD.


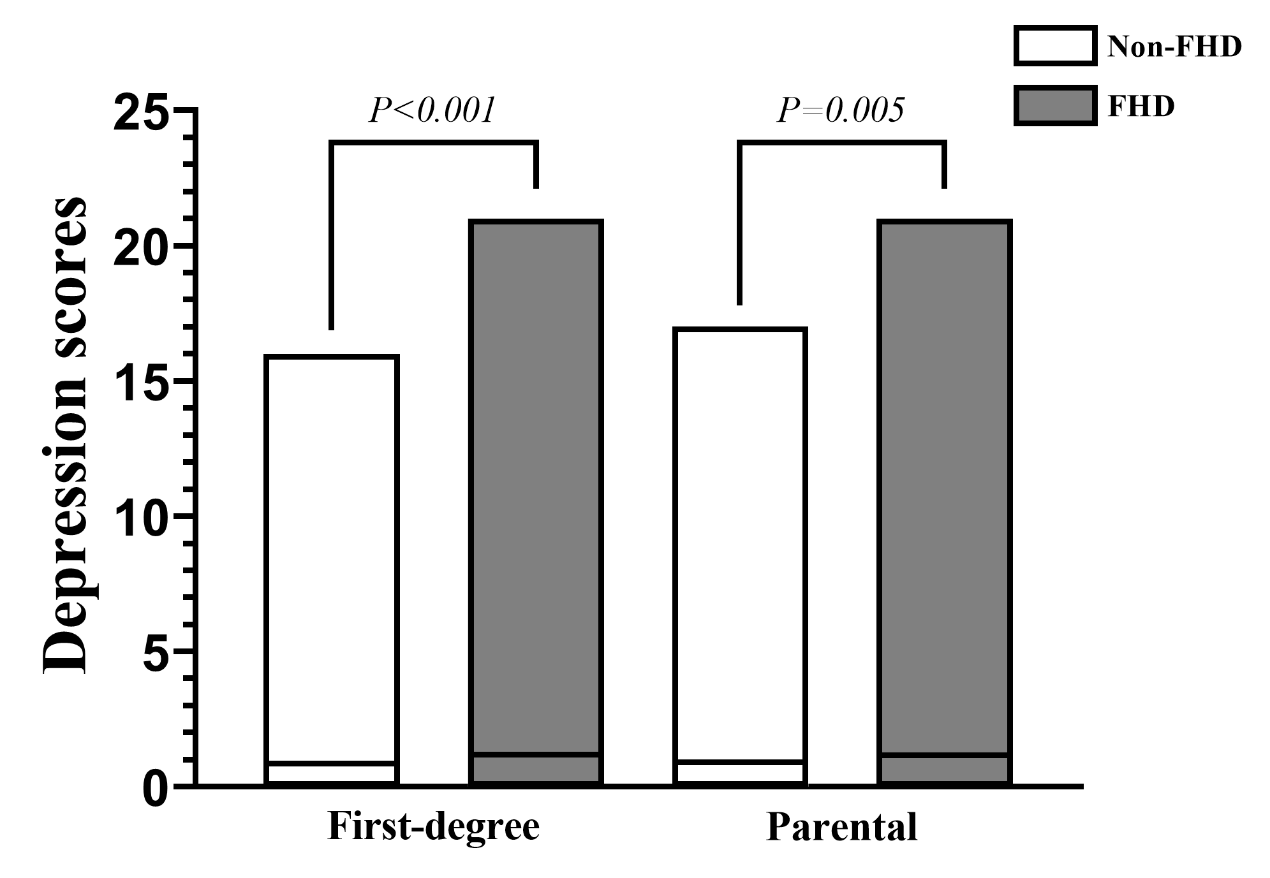


Table S1. Association between the parental family history of diabetes and depressive symptoms.

|  | OR | 95% CI | P value |
| --- | --- | --- | --- |
| Model 1 | 1.242 | 1.062-1.451 | 0.007 |
| Model 2 | 1.241 | 1.056-1.457 | 0.009 |
| Model 3 | 1.237 | 1.053-1.454 | 0.010 |
| Model 4 | 1.248 | 1.061-1.469 | 0.008 |

^1^Abbreviation: OR: odds ratio; 95% CI: 95% confidence interval. ^2^P-values were determined using logistic regression. ^3^Model 1: Unadjusted. Model 2: Adjusted for gender, age and socioeconomic factors (marital status, living alone, employment, and education). Model 3: Adjusted for gender, age, socioeconomic factors and lifestyle risk factors (current smoking, excess alcohol intake, physical inactivity, sedentary behavior, and low dietary score). Model 4: Adjusted for gender, age, socioeconomic factors, lifestyle risk factors, and cardiometabolic risk factors (central obesity, hyperglycemia, hypertension, and dyslipidemia).
